# Patient experience in co-production of care: perceptions about patient safety protocols*

**DOI:** 10.1590/1518-8345.3352.3272

**Published:** 2020-06-01

**Authors:** Diovane Ghignatti da Costa, Gisela Maria Schebella Souto de Moura, Simone Silveira Pasin, Francis Ghignatti da Costa, Ana Maria Müller de Magalhães

**Affiliations:** 1 Universidade Federal de Santa Catarina, Centro de Ciências da Saúde, Florianópolis, SC, Brazil.; 2 Hospital de Clínicas de Porto Alegre, Porto Alegre, RS, Brazil.; 3 Universidade Federal do Rio Grande do Sul, Escola de Enfermagem, Porto Alegre, RS, Brazil.

**Keywords:** Patient Safety, Patient Satisfaction, Patient Participation, Quality of Health Care, Patient-Centered Care, Hospitals, Segurança do Paciente, Satisfação do Paciente, Participação do Paciente, Qualidade da Assistência à Saúde, Assistência Centrada no Paciente, Hospitais, Seguridad del Paciente, Satisfacción del Paciente, Participación del Paciente, Calidad de la Atención de Salud, Atención Dirigida al Paciente, Hospitales

## Abstract

**Objective:**

to analyze the experience of the patient during hospitalization, focusing on the co-production of care related to patient safety protocols.

**Method:**

qualitative study, whose data were collected through the triangulation of multiple sources: document analysis, observation of 10 professionals in the provision of care and 24 interviews with patient-families from 12 clinical and surgical inpatient units of a hospital. Thematic analysis was carried out, based on the concept of co-production.

**Results:**

safety protocols according to the experience of the patient portrayed the role of patient-families as co-producers of safe care. It was found an alignment between perceptions of the patients, institutional definitions and basic national and international patient safety protocols. However, these protocols are not always followed by professionals.

**Conclusion:**

co-production was perceived in the protocols for safe surgery and prevention of injuries resulting from falls. In patient identification, hand hygiene and medication process, it was found that co-production depends on the proactive behavior of patient-families, as it is not encouraged by professionals. The research contributes with subsidies to leverage the participation of the patient as an agent of their safety, highlighting the co-production of health care as a valuable resource for advancing patient safety.

## Abstract (pt)

**Objetivo:**

analisar a experiência do paciente durante a internação hospitalar, com foco na coprodução de cuidados relacionados aos protocolos de segurança do paciente.

**Método:**

estudo qualitativo, cujos dados foram coletados por meio da triangulação de múltiplas fontes: análise documental, observação de 10 profissionais na prestação de cuidados e 24 entrevistas com pacientes-famílias de 12 unidades de internação clínicas e cirúrgicas de um hospital. Procedeu-se à análise temática, ancorando-se no conceito de coprodução.

**Resultados:**

protocolos de segurança segundo a experiência do paciente retrataram o papel dos pacientes-famílias como coprodutores do cuidado seguro. Constatou-se alinhamento entre as percepções dos pacientes, as definições institucionais e os protocolos básicos de segurança do paciente, nacionais e internacionais. No entanto, esses protocolos nem sempre são seguidos pelos profissionais.

**Conclusão:**

a coprodução foi percebida nos protocolos de cirurgia segura e de prevenção de lesões decorrentes de queda. Na identificação do paciente, higienização de mãos e processo de medicação, verificou-se que a coprodução depende do comportamento proativo de pacientes-famílias, pois não é estimulada pelos profissionais. A pesquisa contribui com subsídios para alavancar a participação do paciente como agente de sua segurança, destacando a coprodução de cuidados em saúde como valioso recurso para o avanço na segurança do paciente.

## Abstract (es)

**Objetivo:**

analizar la experiencia del paciente durante la hospitalización, centrándose en la coproducción de la atención relacionada con los protocolos de seguridad del paciente.

**Método:**

estudio cualitativo, cuyos datos fueron recolectados a través de la triangulación de múltiples fuentes: análisis de documentos, observación de 10 profesionales en la atención y 24 entrevistas con pacientes-familias de 12 unidades de hospitalización clínica y quirúrgica de un hospital. Se realizó un análisis temático, basado en el concepto de coproducción.

**Resultados:**

los protocolos de seguridad de acuerdo con la experiencia del paciente retrataron el papel de las familias de pacientes como coproductores de atención segura. Se encontró una alineación entre las percepciones de los pacientes, las definiciones institucionales y los protocolos básicos nacionales e internacionales de seguridad del paciente. Sin embargo, estos protocolos no siempre son seguidos por profesionales.

**Conclusión:**

la coproducción se percibió en los protocolos de cirugía segura y prevención de lesiones resultantes de caídas. En la identificación del paciente, la higiene de las manos y el proceso de medicación se descubrió que la coproducción depende del comportamiento proactivo de las familias de pacientes, ya que no es alentada por los profesionales. La investigación contribuye con subsidios para aprovechar la participación del paciente como agente de su seguridad, destacando la coproducción de la atención médica como un recurso valioso para avanzar en la seguridad del paciente.

## Introduction

For more than a decade, the World Health Organization (WHO) has warned of the need to promote safer practices in the healthcare environment. In the 2008-2009 work plan of the World Alliance for Patient Safety, there is an action that is the focus of this study, which requires attention from health service providers and managers, called Patients for Patient Safety, whose main purpose is to ensure that the patient’s voice is the foundation of the movement for safety^(^ 1 ^)^. At the national level, this action makes up one of the axes of the National Program for Patient Safety (Programa Nacional pela Segurança do Paciente, PNSP), instituted by the Ministry of Health, which describes actions for the involvement of the patient in their safety^(^ 2 ^)^.

Considering the direction of health policies towards improvements in patient safety, through their participation, the focus of this research was on the experience of the patient in the co-production of care, from the perspective of quality in hospital service. The interrelation of the conceptual basis presented in this statement reflects the dynamic and interactive nature of the investigated object.

Among the concepts, it is based on patient engagement in the assessment of health quality^(^ 3 ^)^ and patient safety, consisting of reducing the risk of unnecessary damage associated with health care to an acceptable minimum^(^ 4 ^)^. The concept of patient experience is also emphasized, which involves interactions, organizational culture and patient perceptions throughout the *continuum* of care^(^ 5 ^)^.

In line with these conceptions, co-production is integrated, originating in the Marketing Services area, whose application can be transversal to different areas of knowledge. The classic concept of co-production refers to a process in which the user is considered an inherent part of the production of a given service, so that the result depends on a joint effort between providers and users^(^ 6 ^)^.

In the health field, service delivery occurs through the presence of the actors involved in service meetings. When this characteristic is not recognized, limitations on the success of partnerships between patients and professionals are incurred, with a view to improving care^(^ 7 ^)^. An example of these limitations is found in a recent publication, in which the low quality of care was associated, among other factors, with the lack of co-production in the provision of health care, considering patients and families^(^ 8 ^)^.

Despite several initiatives, there is a long way to go in favor of patient safety, especially in strategies that consider their involvement in the identification of weaknesses in the health system, which incur health care^(^ 9 ^)^. Patient participation in safety is still deficient in clinical practice and systematic actions are needed to create a safety culture in which patients are seen as partners^(^ 10 ^)^.

A multi-center study concludes that patients provide information that portrays the care experienced and, therefore, can contribute with what needs to be changed to improve patient safety and experience^(^ 11 ^)^. Scope review about patient engagement in improving hospital services, shows that there is a lack of research on this theme, pointing to the need for future research with a behavioral focus on patient engagement^(^ 12 ^)^.

These findings from the foray into the literature mobilized the following research question: In the experience of the patient seen in hospitalization units, do perceptions emerge about the actions related to patient safety? The aim of the study was to analyze the experience of the patient during hospitalization, focusing on the co-production of care related to patient safety protocols.

## Method

The research is part of the qualitative aspect, a design recommended to explore and describe the object under investigation, which applies to the study of the perceptions and interpretations that the subjects produce, making it possible to unveil meanings of the studied phenomenon^(^ 13 ^)^. It was anchored in the conceptual framework of co-production^(^ 6 ^)^, which implies the active participation of the patient in their care.

Presearch developed in a public, university, Brazilian hospital, certified by the Joint Commission International. The participants were 22 patients and eight adult family members admitted to 12 clinical and surgical units and 10 professionals who worked directly on patient care, including three nurses, six nursing technicians and an occupational therapist.

The sample was intentional, considering the planning to interview two patient-families *per* unit, totaling 24 interviews and, also, to accompany the professionals available in the care opportunities carried out in the period stipulated for observation. It is noteworthy that six patients attended by their family members participated in the interviews, being considered an interview for the group called patient-family. In two interviews, only the family participated, considering the characteristics of the patients and, in 16, there was the exclusive participation of the patients. Data saturation has guided the decision that enough has been achieved^(^ 14 ^)^.

The inclusion criteria for patients consisted of a hospitalization period of six days or more, interest in sharing their experience and clinical conditions to travel to the interview place, in the unit itself. Regarding the families of the participating patients, in addition to the interest in reporting their perceptions, subjects who have followed the hospitalization period for the most part were included. This criterion was defined, considering the perspective of the participation of the family member in relation to the provision of care. The selection of patient-families followed the census list of the unit, containing the patients’ names and date of hospitalization, addressing the first male patient and the first female patient *per* unit. All patients-families invited to participate accepted and remained in the study. The exclusion criterion adopted was some difficulty in communication, due to an impairment that prevents the participant from verbalizing their perceptions.

In relation to the health team professionals, those who were on active staff and who were assigned to one of the four shifts scheduled for observation in the two selected units were included. Two professionals refused to participate, as they did not want to be accompanied during care. Employees on probationary terms or on fixed-term contracts were excluded. The selection of units to perform the observation occurred after the secondary data collection step, described below.

Secondary data, for document analysis, were extracted by the principal researcher of the Management Information System and the Operational Management System, between January 2016 and October 2018. Institutional documents were found that guide the care routines, described in the form of care policies and plans, selecting those that contained the explicit description of the patient-family involvement in care. In addition to these, results were collected from four quality and safety indicators (hand hygiene, patient identification, falls and patient satisfaction) from the 12 units of the study scope.

The analysis of the indicators defined the units to be observed, choosing the unit that presented the most critical results and the one that obtained the best results in relation to the defined goals. Unit 8 obtained the most critical results in the four indicators and units 1, 4 and 11 obtained the best results in two indicators each. Thus, units 8 and 11 were defined for the observation stage, the latter being defined by drawing among those with the best results.

The primary data collection included field observation and interviews based on a semi-structured script, which were carried out in November 2018. It was opted for passive observation, performed by a member of the research group, with insertion in the investigated object and trained for the technique. Service meetings between users and professionals were the focus of observation, recorded in a script with the following items: behaviors, dialogues, description of the place and activities performed, as well as impressions of the observer. 30 situations that characterize service meetings were followed, totaling 16 hours.

The interviews were audio recorded, conducted by the principal investigator and conducted based on the Critical Incident Technique^(^ 15 ^)^, lasting 30 to 40 minutes. This technique allows to explore and describe the perspectives of the interviewees on significant situations experienced. Thus, it enables the understanding of behaviors, situations and consequences, in order to support the planning of actions, according to what is intended to be achieved through its application.

In the conduct of the interviews, the perceptions that composed the memories of the patients were considered as a critical incident, in which it was possible to co-opt a situation, the present behaviors and their consequences, both positive and negative. The technician guides the memories to be stimulated by means of an initial statement - *Think how was the service in relation to the care related to your safety and your involvement in this care.* After the initial statement, it was waited for the necessary time to remember a significant situation experienced. Then, the dialogue between interviewer and participant was guided by a question script, according to some examples: What situation did you remember? Which people were involved? What did you notice in the behaviors of those involved, including yours? Why was this event selected by you? What could have been different?

After a literal transcription of the primary data from the interviews and observations, thematic analysis began jointly, with the support of the Nvivo 11 software, to organize the analysis *corpus* ^(^ 13 ^)^. Thus, the themes were identified and grouped for the composition of the category, according to the stages of pre-analysis, exploration of the material, treatment of the results obtained and interpretation.

The survey was approved under the CAEE number: 01092918.2.0000.5327, with a term for the use of institutional data and terms of free and informed consent. In the presentation of the results, the interviewees’ statements were coded, using the letters P and F, to refer to the participation of the Patient and the Family, respectively, followed by a number according to the chronological order of the interviews. The observations were coded by the letter O, followed by a number, according to the chronological order of completion. The method followed the criteria indicated for qualitative research, described in the check list COREQ^(^ 16 ^)^.

## Results

As a characterization of the participating patients, half were male, a result expected in the planning, according to the inclusion criteria. The median age was 57 years old, with a minimum age of 34 years and a maximum age of 75 years. The length of hospital stay corresponded to a median of 14 days of hospitalization, with a minimum of six and a maximum of 52 days. Regarding the level of education, 18 had elementary and high school, five was under graduated and one was illiterate. The reasons for hospitalization were related to clinical and surgical comorbidities, both acute and chronic.

The results emerging from observation and interviews were grouped into three categories and in this study, the topics in the category of safety protocols were approached according to the experience of the patient: patient identification, safety in medication administration, care for the prevention of injuries resulting from falls, care for infection prevention with a focus on hand hygiene, consent process, safe surgery and care to prevent pressure injury. As a cross-sectional empirical result, the role of patient-families as co-producers of safe care stands out, based on the integrated analysis of information derived from documents, observations and interviews.

The use of a patient identification bracelet was observed in the situations observed and in the interviews. This care is recognized and valued by patients, as highlighted in the participants’ statements: *[...] my bracelet is my real identification, it has my name, it has a code [...]* (P2); *[...]I know what this bracelet is for, it has my name, it means that this is a first-class hospital* (P7).

According to the Patient Identification Policy and Plan, there are mandatory moments for checking the identification, as well as the description of how to do it, according to the care provided, with the inclusion of the patient and family. The manifestations and observations marked out how the identification of the patient occurs and at what times. One of them concerns the collection of exams, the care of which was perceived by the patients, according to the following example: *[...] I collect blood every day at 7 am and they use my bracelet at this time, look at the bracelet and then look at the papers and bottles that bear my name* (P1).

Despite the recognition of the need for a conference, when investigating co-production in this care, as another security barrier, it was found that it still does not occur: *[...] when collecting exams, everyone is very careful, the bottles are all identified, but I’m not at liberty to look if it’s right, but I trust they don’t make mistakes* (P2).

Patients also realized that there is a concern with their identification in moments of transition of care, when they are transported to other areas of the hospital: *[...] when I went to surgery, they looked at the bracelet and the papers to confirm that it was me, only after they took me* (P21)*.*

Another mandatory moment for the team to check the identification of the patient, according to the documents that guide the routine, is in the administration of medications, an action considered essential for the safety of the medication process. It was found that, according to the experience of the patients, there is a conference before the administration of drugs. *[...] when they come to the room, they look at the bracelet at the time of medication, I think this is very important. I notice that the technicians read the medication in the spreadsheet, make sure it is the right one, so that there is no problem of giving the wrong medication* (F9).

In the observations, it was possible to monitor nursing technicians in nine situations involving medication administration, noting that there was an identification check in two (O1 and O12). In situations where the professionals did not carry out the conference, it was possible to verify that there was a relationship of trust established between the professional and the patient, evidenced by dialogues between them, calling themselves by name, interacting with each other during care, in a relaxed environment and quiet (O2, O3, O4, O13, O17, O18, O22).

We also sought to deepen the active role of patient-families in the administration of medicines, if they received guidance on this, as they do. The manifestations were of ignorance of this care by some patients, as illustrated by the statement: *[...] I even laugh at the girls, because they come to look at my bracelet if I have been here for more than 50 days, I say, do they not know who I am? [...] I don’t know, and I didn’t ask why they do that either* (P8).

On the other hand, it was possible to verify that there are patients-families engaged in the treatment, in order to follow the actions of the nursing team in relation to the medication process, being co-producers: *[...] what has happened is that I take a medicine that has a different dose on even days and odd days and I always need to confirm the dose, because it often comes wrong* (P20).

The observations complement the findings, in the sense of demarcating other situations not verbalized by the patient-families, but which are included in the institutional reference documents, as recommended to proceed with the identification check. In the following observations, there was no verification of the identification bracelet when approaching patients: changing the central catheter dressing (O7), evaluating the general conditions of the patients (O8, O10, O14, O15) and performing the physical examination on the patient, focusing on the abdomen (O11). However, this care was verified in six situations in which professionals assessed the general conditions of the patient, including guidance on the use of the identification bracelet for care (O25, O26, O27, O28, O29, O30).

Another safety protocol that emerged from the interviews and observations, concerns the preventive measures for injuries resulting from falls, mentioned in the analyzed documents. According to the documents, adult patients are systematically assessed for the risk of falls, using an evaluation scale that results in a final score. Preventive measures are implemented for patients who are at high risk of falling, including identification with a yellow bracelet. In the interviews, it was found that several patient-families are aware of why they wear this bracelet, as shown in the following example: *[...] the yellow bracelet is anti-fall. It needs to keep the bed always closed [with rails], with both sides closed so you don’t fall and always be with a companion to take him to the bathroom, do things with him* (F3).

Among the interviewees who wore the yellow bracelet, only one was unaware of what care he would need to have; however, he knew why he used such a bracelet and perceived the professionals’ attention to it: *[...] when they put on the bracelet, they said it was for risk of falling and that was it. I noticed that where I went for exams, they looked at my bracelet and were very careful with me, to sit on the bed, to put me in the chair* (P8).

Regarding the preventive measures of falls observed, it was found that the professionals, when finishing the service, repositioned the bed in the low position and raised the bed rails (O7, O16, O18, O19). It is also possible to follow the reinforcement of the guidelines on care to prevent falls, emphasizing the high railings, the low bed and the request for help to get out of bed accompanied (O26, O27, O28, O29).

The participants highlighted the care related to hand hygiene, including guidance for patients-families, with recognition of the information materials available in the rooms. *[...] both at the entrance and at the exit [from the room] I think it’s very important, it doesn’t bring anything from the street to the person and it doesn’t take anything from inside to the street* (P13).

Similar to other care, there was also a manifestation regarding the lack of hand hygiene, denoting that patients are attentive, despite showing passivity when they observe that care was not followed: *[...] whoever enters the room here uses alcohol gel at the entrance and exit, although not all, but many use it* (P7).

In the 30 situations observed, hand hygiene was performed in 16, of which seven were administered medication (O1, O2, O3, O9, O12, O17, O18, O22), a diaper change (O16), two physical exams (O11, O26), handling of enteral tube (O27) and in the general assessment of the patient (O25, O28, O29, O30). It was possible to verify that, when entering the room for general assessment of the patient or for some guidance, without direct physical contact, the professional did not follow the practice of hand hygiene (O5, O6, O7, O9, O14, O15, O20). Furthermore, in some procedures it was also not observed, such as when changing the dressing of a central catheter (O7), bed bath (O21), mechanical restraint (O24), administration of oral medications (O4, O13) and handling of enteral tube (O19, O23).

It was also possible to count on the participation of patients who had experiences with surgical interventions, whose reports portray the care recommended in the institutional documents regarding the consent process and safe surgery. This care was expressed by all respondents who had this experience, as shown in the following example: *[...] in my surgery everything went well [...]. They informed me, explained me, they checked the knee I had to operate, they put a mark here, because we know that the sides have already changed, but this cannot happen* (P21).

Regarding the pressure injury prevention protocol, there were no reports in the interviews, but it was possible to follow the guidance given to a patient’s family member, during the bed bath (O21). The professional explained about the importance of skin care for the patient, about hydration, demonstrating the care throughout the bath, interacting with the patient and his family.

## Discussion

The assistance guidelines analyzed are in line with the global challenges proposed by WHO more than a decade ago. The first challenge directs care to prevent infections through hand hygiene^(^ 17 ^)^. The second, deals with safety barriers for harm reduction associated with surgical interventions, including the participation of the patient-family in the consent regarding the type of planned procedure, confirmation of the surgical site and verification of the identification of the patient before anesthetic induction^(^ 18 ^)^. The third, more recently launched, aims to reduce preventable adverse events related to the medication process^(^ 19 ^)^.

The safety protocols identified refer to international targets for patient safety. These goals guide barriers to the occurrence of adverse events in situations of higher risk, involving patient identification, communication in the care environment, medication process, hand hygiene, surgery and fall prevention^(^ 20 ^)^. In addition to this care, patient-families also expressed perceptions about care related to transfer between sectors^(^ 2 ^)^.

Results of research that assessed the impact of Accreditation programs on Brazilian health organizations, highlighted greater patient involvement when the institution has Accreditation status^(^ 21 ^)^. In line with this result, in this research it was found that patient-families demonstrated knowledge about the purpose of patient identification, whose meaning of such practice, in the view of patient-families, is equivalent to health care in first world countries.

Regarding medication administration, perceptions of the patients showed fragility in the process, considering the important safety barrier of checking identification before the medication administration, which is only followed by some professionals. It was found that there is variability in the behavior of professionals, compared to the standard of quality and safety defined by the institution. In addition, it was found that the patient considers the way medication administration takes place, when there is a relationship of trust established with the care team, making identification checking unnecessary.

The concern with correct identification, as an important barrier for the prevention of adverse events, has been published since the theme became a global issue, as a result of the initiatives of the World Alliance for Patient Safety^(^ 1 ^)^. A study evaluated the use of the identification bracelet in patients in the same field, at the time they were made manually by nursing professionals, found that 11.9% of patients had the identification bracelet with errors, with incomplete names, record numbers differences, lack of data legibility and problems with integrity^(^ 22 ^)^.

Still in the same scenario, another study found an increase in the rate of adherence of professionals to verify the identification of the patient before the highest risk care after changes in the process, with the inclusion of computerized labels for making the bracelets, sensitizing the teams^(^ 23 ^)^. A study carried out at another university hospital, also in the south of the country, found that adherence to patient identification is deficient, considering that, in 71.6% of the analyzed cases, patients were identified, and the authors considered that the result should be close to 100%^(^ 24 ^)^. Research carried out in Turkey, in a JCI certified hospital, the results were alarming, in relation to the lack of knowledge of the patient about the use of the identification bracelet, as well as of the professionals about when it is essential that the conference is held, so that the care provided is safe in that concern^(^ 25 ^)^.

Regarding the involvement of patients in safety actions, it was found that co-production for patient safety is still incipient, when the participation in the mandatory moments of checking their identification is analyzed, in the sense of depending on the patient’s inherent posture. If the patient-families are proactive and knowledgeable, it was found that they feel free to participate, getting involved about which medications they are receiving, checking the identification labeled on the medication. However, it was not identified that there is a stimulus of this practice, on the part of professionals, in the production of care and the relationship of trust that patients place in professionals is considered sufficient by patient-families so that there are no failures.

One study, which sought feedback from patients on safety, found that 35% of the reports were classified as a patient safety incident, with the most frequent incident being the medication error, which was present in the speeches of one in 10 interviewed patients^(^ 11 ^)^. Research that analyzed the preparation and administration of drugs in the same field in which this research was developed found that one of the current problems concerns the verification of patient identification at the time of administration, since it is performed only with the visual resource of the professional, not counting with technology support, such as bar code reader^(^ 26 ^)^.

In Canadian research, an innovative visual tool on patient safety was proposed, guiding postures and behaviors so that they participate in the care, which is available in the users’ circulation environments. The assessment of the tool by patients and family members indicated that they felt more confident to ask questions of professionals, by stimulating the materials used^(^ 27 ^)^.

In addition to finding strategies to influence patients to ask questions, in order to promote their involvement in the safety of their care, it is also necessary to prepare professionals for this participation. In this sense, a study analyzed government policies and programs in five countries, and in Canada, the actions were directed towards monitoring patient engagement, stating the need to invest in the training of professionals, through education programs, to develop co-production strategies in healthcare^(^ 28 ^)^. Other results demonstrate barriers to be overcome in this sense, due to the lack of skill of the health team and cultural issues present in the hospital service environment related to the lack of knowledge about how the patient can collaborate with safety^(^ 12 ^)^. In addition to cultural differences, the reduction of gaps in communication between patients-families and teams requires time organization, for investment in such a strategy^(^ 29 ^)^. The education of professionals and patients for co-production in health is highlighted as a need to train agents of change in this context^(^ 7 ^)^, becoming urgent due to the occurrence of preventable adverse events related to health care^(^ 9 ^)^.

Another protocol described in PNSP^(^ 2 ^)^ is consolidated in the documents and care practices of the studied field, through a fall prevention plan, whose care was present in the perceptions of patients-families study participants and the behavior of professionals on a constant basis. The results demonstrate that professionals encourage the patient and family to co-produce care in service meetings, in relation to preventive measures during hospitalization. In a national study about this care, there are no aspects on the involvement of the patient and family in the prevention of falls^(^ 30 ^)^. On the other hand, we found the description of 11 actions to prevent falls, with the participation of the patient and family in it^(^ 31 ^)^. The implementation of an intervention, to promote the participation of the patient and family in the fall reduction plan, resulted in a significant reduction in falls in general and falls with damages^(^ 32 ^)^. Studies demonstrate that patient and family involvement in care is relevant, which must be implemented systematically in hospital health services.

Another action related to patient safety, marked in the experiences of patients who participated in the research, concerns hand hygiene. The reports demarcated recognition of the guidelines, informative materials available at the institution, with apprehension about the mandatory moments to proceed with hand hygiene, both in relation to their own posture, and through attentive observation of the behavior of the professionals regarding this care. However, although the patient-family demonstrates conditions to actively participate in their safety in relation to hand hygiene, as it is an action that draws their attention, they demonstrated passivity when care is not followed by professionals. Co-production in the provision of care, in this sense, did not occur, considering that the adopted behavior was expectant in the face of the situation.

In view of the results presented and the findings in the literature, contributions to the advancement of co-production in care are verified, by signaling the *status quo* of the participation of the patient as an agent of safety actions in their care. Another aspect that arises from this incursion concerns the space for co-production to be promoted by the health team, for the insertion of the patient as the central axis of safe care, a condition that demands in-service education to implement such a strategy. With this, it is expected to promote the production of knowledge for health and have repercussions on the processes of professional training, health care and management.

Regarding the limitations of the study, it is considered that the interviews, since they were carried out during the hospitalization period, may not have contemplated the entire experience lived during the hospitalization. Another issue refers to the possible fear of participants expressing delicate situations, while they still depend on the assistance that is offered to them, which may have influenced the content of the reports.

## Conclusion

It is possible to verify an alignment between the perceptions of the patients, the institutional definitions of the studied field and the basic protocols described in the National Program for Patient Safety. However, although these basic protocols are part of the perceptions of patient-families, they are not always followed by professionals, incurring risks for the safety of care, when important safety barriers are not remembered or ignored. It is also noted that this behavior was perceived by the patient-families, with no warning signs on the part of them to the professionals, a condition that indicates fragility in the care process, due to the lack of active participation, mainly in the patient identification conference mandatory moments and hand hygiene.

With that, it was observed that co-production for patient safety is still incipient in relation to this care. Despite the fact that patients-families show the potential to co-produce, the professionals did not stimulate this practice.

The relationship of trust between patients and professionals, although it is a positive factor in the hospital environment, interposes itself as fragility to safety, when it justifies the passive attitude of patients-families towards care, as they consider that there will be no failures in the face of such relationships.

In the experience of patients undergoing surgery, co-production was present, at the time of consent and marking of the surgical site, when the intervention required laterality. Also, there was a stimulus for co-production of care related to the prevention of falls. Co-production for safety in drug administration was found to depend on the proactive behavior of some patient-families. Its initiative in checking and asking questions led professionals to adopt a favorable attitude towards co-production, stimulating interaction through clarifications, enabling the patient to check the type of medication, dose, time and identification.

The research contributes with subsidies to leverage the participation of the patient as an agent of safety actions in their care. Therefore, it was considered that the co-production of care oriented to patient safety is a valuable resource for advances in favor of patient safety. Furthermore, co-production is a viable solution for health services that aim to continuously improve care practices, with a view to developing effective partnerships between health teams and patient-families, for the benefit of patient safety.
